# The Insecticidal Efficacy and Physiological Action Mechanism of a Novel Agent GC16 against *Tetranychus pueraricola* (Acari: Tetranychidae)

**DOI:** 10.3390/insects13050433

**Published:** 2022-05-05

**Authors:** Yanyan He, Guangzu Du, Shunxia Xie, Xiaoming Long, Ganlin Sun, Shusheng Zhu, Xiahong He, Yixiang Liu, Youyong Zhu, Bin Chen

**Affiliations:** 1 School of Agriculture, Yunnan University, Kunming 650500, China; heyanyan0505@outlook.com; 2 State Key Laboratory of Conservation and Utilization of Biological Resources of Yunnan, College of Plant Protection, Yunnan Agricultural University, Kunming 650201, China; duguangzu1986@163.com (G.D.); xieshunxia2022@163.com (S.X.); longxm6688@163.com (X.L.); ganlin678@163.com (G.S.); shushengzhu79@126.com (S.Z.); hexiahong@hotmail.com (X.H.); liuyixiang@ynau.edu.cn (Y.L.)

**Keywords:** GC16, insecticidal activity, action mechanism, *Tetranychus pueraricola*

## Abstract

**Simple Summary:**

Spider mite is major pest in agriculture and have developed resistance to commonly used pesticides. Therefore, it is urgent to discover new pesticides to control the pest. In order to provide alternatives for its management, we evaluated the effectiveness of a new agent GC16 against the spider mite *Tetranychus pueraricola.* Then, we preliminarily revealed the its acaricidal mechanism of action based on the damage of cuticle and organelles of mites. We confirmed that GC16 has a good controlling effect on *T. pueraricola* and it is not harmful to *Picromerus lewisi* and *Harmonia axyridis*. Our research provides not only an alternative pesticide for the management of spider mites, but also guidance for the application of GC16 in sustainable agriculture.

**Abstract:**

Chemical control plays a crucial role in pest management but has to face challenges due to insect resistance. It is important to discover alternatives to traditional pesticides. The spider mite *Tetranychus pueraricola* (Ehara & Gotoh) (Acari: Tetranychidae) is a major agricultural pest that causes severe damage to many crops. GC16 is a new agent that consists of a mixture of Calcium chloride (CaCl_2_) and lecithin. To explore the acaricidal effects and mode of action of GC16 against *T. pueraricola*, bioassays, cryogenic scanning electron microscopy (cryo-SEM) and transmission electron microscopy (TEM) were performed. GC16 had lethal effects on the eggs, larvae, nymphs, and adults of *T. pueraricola*, caused the mites to dehydrate and inactivate, and inhibited the development of eggs. GC16 displayed contact toxicity rather than stomach toxicity through the synergistic effects of CaCl_2_ with lecithin. Cryo-SEM analysis revealed that GC16 damaged *T. pueraricola* by disordering the array of the cuticle layer crest. Mitochondrial abnormalities were detected by TEM in mites treated by GC16. Overall, GC16 had the controlling efficacy on *T. pueraricola* by cuticle penetration and mitochondria dysfunction and had no effects on *Picromerus lewisi* and *Harmonia axyridis*, indicating that GC16 is likely a new eco-friendly acaricide.

## 1. Introduction

Sustainable agriculture focuses on producing long-term crops with minimal effects on the environment and is being watched around the world [1,2,3]. Biodiversity is an important part of sustainable agriculture [3,4]. Considering the damage to biodiversity caused by conventional pesticides, there is a need to develop new alternative and eco-friendly pesticides to protect biodiversity [5].

Spider mites are major pests worldwide that cause serious damage to many crops and vegetables and great economic losses in agriculture and horticulture [6,7,8,9,10]. For a long time, people have mainly and widely relied on chemical pesticides to control mites. However, because of the overuse and misuse of pesticides, an increasing number of reports from different countries have revealed that spider mites have developed different degrees of resistance or cross-resistance to a variety of pesticides, including the global top-selling acaricide abamectin [8,9,11,12,13,14]. Unfortunately, the development of resistance has led to the increased use of chemicals. Therefore, pesticide residue and pest resurgence have appeared in ecosystems and are threatening agriculture and human health [15,16]. Ecological (eco-) pesticides are good alternatives to synthetic chemical pesticides in sustainable agriculture. GC16 (a mixture of natural botanical extracts and other inorganic nutrients) is promising to be developed as an ecopesticide.

GC16, composed of Calcium chloride (CaCl_2_, 45%) and lecithin (55%, extracted from soybeans), was recently found to have the ability of killing pests. GC16 has not been registered and is still being tested in a field trial across 15 provinces in China, including Beijing, Heilongjiang, Hainan, and Yunnan [17]. CaCl_2_, an inorganic salt, can be used as a plant nutrient or as an additive or firming agent in general [18,19,20]. Lecithin is a food-grade substance that is safe for human health and has been evaluated as a food additive by the Joint FAO/WHO Expert Committee on Food Additives (JECFA) (https://apps.who.int/food-additives-contaminants-jecfa-database/Home/Chemical/1477 (accessed on 1 April 2022)). As an edible and digestible surfactant, antioxidant, and emulsifier of natural origin, lecithin has been widely used in the food, cosmetics, and pharmaceutical industries, especially for margarines and in chocolate [21,22,23,24,25]. Studies have shown that lecithin enhances the thermal tolerance of fish and protects them against cellular stress after exposure to organochlorine pesticides [26]. Additionally, lecithin can be used in carriers for drug delivery [27]. Moreover, lecithin was reported to decrease fungicide residue [28] and this compound along with its derivatives could be applied for post-harvest treatment because they can protect fruits and vegetables from stress-related damage and improve their quality during storage and shelf life [29]. Lecithin has been mainly used as a food additive, surface-active agent, or emulsifier of pesticides, but its insecticidal potential as a major component has not been evaluated, and the mechanism of action of CaCl_2_ + lecithin coapplication to control pests has not yet been revealed.

*Tetranychus pueraricola* (Ehara & Gotoh) (Acari: Tetranychidae) is a newly reported spider mite that is often found in China [30]. It has become the second most frequently sampled *Tetranychus* spider mite in China with 21.2% frequency, compared with *T. truncatus* (48.5%) and *T. urticae* (red) (5.7%) based on a long-term survey from 2008–2017 [10]. However, *T. pueraricola* is not a recent invasive pest but rather a long-standing species in China [30]. This species was first described as a new species by Ehara and Gotoh (1996) [31], is close to the two-spotted spider mite *T. urticae* Koch (red form) [7,32], and has long been misidentified as *T. urticae* (red form), *T. cinnabarinus,* or even *T. truncatus* [30].

This work is the first to evaluate the insecticidal efficacy and explore the mechanism of action of GC16 by using the spider mite *T. pueraricola* as a model species through bioassays, cryogenic scanning electron microscopy (cryo-SEM), and transmission electron microscopy (TEM) as the technological means.

## 2. Materials and Methods

### 2.1. Insects and Pesticide

The *T. pueraricola* population was collected in 2021 from *Polygonatum sibiricum* Delar. ex Redoute in a forest of *Pinus kesiya* Royle ex Gordon var. *langbianensis* (A.Chev.) Gaussen in Lancang, Yunnan Province, China (altitude: 1492 m, 22°48′15″ N, 99°47′22″ E). The population was collected by random sampling. The collection points of the eight subsamples were at least 500 m away from each other, and each subsample consisted of at least 500 adult mites. The field population of mites from this area was obtained by mixing the subsamples together. Then, the collected mites were maintained on pesticide-free and insect-free kidney bean plants (*Phaseolus vulgaris* Linn) “Bifeng” in the greenhouse of Yunnan Agricultural University, Kunming, Yunnan Province, China. After three generations of indoor breeding, these mites were used for the following experiments. The mite species was identified by Professor Xiaoyue Hong, a mite classification expert working at Nanjing Agricultural University in China. Based on specimen morphological identification and mitochondrial examination, the mite was confirmed to be *T*. *pueraricola.*

The common natural enemy insects *Picromerus lewisi* Scott (Hemiptera: Pentatomidae) and *Harmonia axyridis* Pallas (Coleoptera: Coccinellidae) were selected as non-target organisms. Non-target organism *P. lewisi* was fed on yellow mealworm and kept in the greenhouse of Yunnan Agricultural University, and *H. axyridis* was bought from Zhongnong Yaxing (Beijing Zhongnong Yaxing Biotechnology Co., Ltd., Beijing, China).

The tested agent GC16 is composed of a mixture of CaCl_2_ (45%) and lecithin (55%). Detailed information is as follows: CaCl_2_ (CAS: 10043-52-4, AR, 96%, Shanghai Macklin Biochemical Co., Ltd., Shanghai, China); lecithin (CAS: 8002-43-5, from soybeans, >98%, Shanghai Macklin Biochemical Co., Ltd., Shanghai, China). Water was used as the solvent when preparing GC16 solutions.

### 2.2. Bioassays

#### 2.2.1. Bioassay of GC16 against Different Stages of *T. pueraricola*

##### Egg Bioassay

The ovicidal toxicity of GC16 to *T. pueraricola* eggs was assessed by the petri dish-spraying method according to a previous study with some modifications [33,34,35,36,37]. In total, 15 adult females were placed on a 20 mm diameter bean leaf disc placed on 0.3% agar in a petri dish for egg deposition. After 12 h, the females were removed, and the leaf discs were evenly and fully sprayed with a power sprayer. After the discs had dried, the eggs were counted, and the petri dishes with the treated leaf discs were placed in an incubator at 25 ± 2 °C with 65 ± 10% RH on a 16:8 (L/D) photoperiod. Five concentrations of GC16 were used to generate a regression equation (0, 1.25, 5, 10, 20 g/L). Each treatment was represented by three replicate leaf discs. The number of emerged larvae was counted daily until all the eggs for control had hatched except died eggs with physiological causes.

##### Larva and Nymph Bioassay

For the bioassays with larvae and nymphs, 20 adult female *T. pueraricola* were placed on leaf discs on 0.3% agar to lay eggs for 12 h [14,35]. Once the larvae/nymphs had emerged, their numbers were counted using a microscope, and the dead larvae/nymphs and other instars were removed. Then, the discs were sprayed with GC16 solutions (six concentrations of GC16; 0, 0.83, 1.67, 3.33, 6.67, 13.33 g/L) according to the same method described above. The leaf discs with larvae and nymphs were dried and stored in the incubator described previously. After 24 h, the living larvae and nymphs were counted.

##### Adult Bioassay

According to Ay (2005) [34], mortality tests were performed before each experiment to determine the range of concentrations that would produce 10~95% mortality. Adult female mites (30~50) were transferred to each bean leaf disc (2 cm diameter) placed in a petri dish, and each dish was covered with a lid containing small holes to avoid condensation of water vapor [14]. All experiments were conducted using three replicates of each concentration of GC16 (0, 0.83, 1.67, 3.33, 6.67, 13.33 g/L). The leaf discs with mites were examined under a microscope to remove the dead and inactive mites and then the discs were sprayed with a power sprayer as described above. After air-drying, the leaf discs were placed back into the plastic petri dishes (3.5 cm diameter) with 0.3% agar to preserve moisture, a method modified according to Xu et al. (2018) [14]. The petri dishes with the treated mites were placed in an incubator described earlier. After 24 h, the numbers of live and dead mites were counted. Mites were recorded as dead if they failed to move when touched with a soft brush. The results were not used if the mortality in the control exceeded 15%.

#### 2.2.2. Bioassays of GC16 by Different Methods

##### Slide-Dip Assay

As recommended by the FAO, 2–3 cm long pieces of double-sided tape were adhered to glass slides [35,38]. The backs (rather than feet or mouthparts) of uniform adult females were gently stuck to the tape using a small brush. There were 45 mites on each slide, and the experiment was repeated three times. The slides were kept in an incubator for 4 h, and the mites were examined with a microscope (SZ51, Olympus, Tokyo, Japan). Dead and inactive individuals were removed. Slides containing mites were dipped into 6.67 g/L GC16 and shaken gently for 5 s. The excess liquid was quickly removed with absorbent paper. The control group was treated in the same way with water. The slides were then placed in a glass petri dish with soaked gauze on the bottom and plastic wrap with a small hole on the top and placed in an incubator as mentioned earlier. After 24 h, the dead mites and live mites were counted. A mite was considered dead if it did not respond to light touch with a small brush. If the mortality for the control exceeded 15%, the trial was repeated.

##### Leaf-Dip Assay

Following the methods of Xu et al. (2018) [14] and Wang et al. (2015, 2016) [13,39], bean leaf discs (2 cm in diameter) were dipped into a 6.67 g/L GC16 solution for 10 s, while leaf discs dipped into water were set as control. The leaf discs were then placed on filter paper to dry. After drying, the leaf discs were backed up and attached to 0.3% agar in a 3.5 cm diameter plastic petri dish. Approximately 25 ~ 30 adult female mites were transferred to each disc, and each petri dish was covered by a cover with small holes to avoid water vapor condensation. The experiment was repeated three times. Petri dishes containing the mites being tested were kept in incubators under the same conditions as those described previously. After 24 h, the numbers of live and dead mites were counted in each petri dish. Mites that did not move after being touched by a soft brush were recorded as dead. The results were not used if the mortality rate for the control treatment exceeded 15%.

##### Spraying Assay

The spraying assay was conducted in a similar manner as the assay in the section *Adult Bioassay* [2]. Approximately 35 healthy adult female mites were transferred to bean leaves, and three replicates were performed. Dead and inactive mites were removed under a microscope. Then, a solution containing 6.67 g/L GC16 was sprayed equally on the bean leaves with the mites with a power sprayer. Then, the plants with the mites were kept in incubators at the conditions described previously. Twenty-four hours later, the numbers of live and dead mites were counted. Mites that did not move after being touched by a soft brush were recorded as dead. Sprayed water was used as a control under the same conditions. If the mortality rate for the control exceeded 15%, the experiments were repeated.

#### 2.2.3. Bioassays for the Different Components of GC16

Bioassays for the different components of GC16 in adult female *T. pueraricola* were performed by the spraying method as described in the previous paragraph with a GC16 concentration of 6.67 g/L. Water was used as a blank control, and the CaCl_2_ and lecithin treatments were administered at concentrations of 3.03 g/L and 3.64 g/L, respectively.

### 2.3. Observation of Poisoning Symptoms for T. pueraricola

To observe poisoning symptoms [40,41,42], 25 mites were carefully transferred to bean leaves. *T. pueraricola* were treated with GC16 at concentrations of 6.67 g/L and 2.00 g/L by the spraying method, and water was used as the control. Observations were made under a microscope (SZ51, Olympus, Tokyo, Japan) at 20 min, 40 min, and then every 2 h from 2 to 24 h; each observation was made with three replicates.

### 2.4. Effects of GC16 on the Morphology of Female Adult T. pueraricola

By the slide-dip method (details described above), 6.67 g/L GC16 was used to evaluate the effects on the morphology of female adult *T. pueraricola*. Treatment with water under the same condition was set as the control. After treatment for 24 h and 48 h, mites were photographed by microscopy (LEICA M205 FA, Wetzlar, Germany), and the body lengths and widths of mites were measured [43]. The relative shrinkage rate (Rst) of length = (body length of control for n h—body length of GC16 for n h)/body length of control for n h (n is the number of hours after treatment; h means hour); the Rst of width was calculated the same.

### 2.5. Effects of GC16 on the Egg Hatching Rate and Developmental Duration of T. pueraricola

Fresh, healthy, and uniform female adult mites were selected and placed on common bean leaves. After 12 h of laying eggs, the adult mites were removed with a brush, and approximately 20 ~ 40 eggs were kept on each leaf [13,35]. The mite eggs were treated with 10 g/L GC16 by the petri dish-spraying method described above, with water as a control, and each treatment consisted of three biological replicates. The eggs were observed and photographed under a microscope (LEICA M205 FA, Wetzlar, Germany) every day, and the hatching rate and development time (time after egg lay) were calculated.

### 2.6. Cryo-SEM (Scanning Electron Microscopy)

The tested mites were treated with water (control), GC16, CaCl_2_, or lecithin. The concentration of GC16 was set to its LC_50_ (2.00 g/L), and the concentrations of CaCl_2_ (0.90 g/L) or lecithin (1.10 g/L) corresponded to the ratio in GC16. After treatment for 24 h, live mites were used for scanning/transmission electron microscopy (SEM/TEM) analysis.

According to Walther (2001) [44], Yu et al. (2011) [45], and Yan et al. (2021a) [46], to avoid chemical fixation and drying artifacts and obtain the most direct and real images of mites in a defined physiological state, a fast frozen technique was used. For cryo-SEM, fresh mites were directly and gently glued to the sample table and frozen in supercooled liquid nitrogen for 2 min. The samples of each replicate were then transferred to a preparation chamber at −140 °C. Next, sublimation was performed at −90 °C for 10 min, followed by coating twice for 60 s each time. The samples were observed and photographed with a ZEISS Sigma 300 scanning electron microscope.

### 2.7. TEM (Transmission Electron Microscopy)

Sample preparation was the same as that described above. A previous method was modified as appropriate [47,48,49,50]. Samples were fixed overnight at 4 °C using 2.5% glutaraldehyde in 0.1 M PB (pH 7.4). Samples were then washed with 0.1 M PB (pH 7.4) three times for 15 min each time. Afterward, the samples were post-fixed with 1% OsO_4_ for 2 h at 4 °C, washed with 0.1 M PB (pH 7.4) three times for 15 min each time, followed by serial ethanol dehydration and acetone transition for 5 min, embedded in Epon 812 resin, and polymerized at 60 °C for 48 h. Serial ultrathin sections with a uniform thickness (60 nm) were made using a Leica EM UC7 ultramicrotome. The ultrathin sections were then loaded onto 50-mesh Cu grids and double-stained with 2% uranyl acetate and lead citrate before observation with a JEM 1400 Plus transmission electron microscope at 120 kV.

### 2.8. The Effects of GC16 on Non-Target Organisms

The target pest mite *T. pueraricola* and non-target organisms *P. lewisi* and *H. axyridis* were treated with 6.67 g/L GC16 using the spraying method mentioned above in the section *Spraying Assay*. The *P. lewisi* and *H. axyridis* were placed in insect-rearing cages (120 mesh, 30 cm × 30 cm × 30 cm) that provided yellow mealworm and aphids, respectively. In total, 20~40 insects were tested each repeat (three repeats each treatment), and insects treated with water in the same way were set as control. Twenty-four hours later, the dead insects were counted and the mortality rate was calculated.

### 2.9. Statistical Analysis

For the developmental stage bioassay data, the slope ± SE, LC_50_ values, 95% fiducial limits, chi-square values, and degrees of freedom (df) were calculated by probit analysis using Polo Plus 2.0 software (LeOra software, Berkeley, CA, USA).

The other data were analyzed using SPSS software, v. 25.0 (SPSS Inc., Chicago, IL, USA). The graphs were created by Sigmaplot 14.0 (Systat Software Inc., San Jose, CA, USA) and grouped by Adobe Illustrator 2021 (Adobe Systems Inc., San Jose, CA, USA). After normality test, the data were in accordance with normal distribution or approximate normal distribution. Differences between the effects of different bioassay methods or different components were analyzed using one-way analysis of variance (ANOVA). Significant differences between treatments were based on Tukey’s honestly significant difference (HSD) test. Differences in body length/width among the different treatments were analyzed by one-way ANOVA with Tukey’s honestly significant difference (HSD) test. Differences in the egg hatching rate and development duration between the two groups were compared using independent sample *t* tests. One-way ANOVA with Tukey’s honestly significant difference (HSD) test was used to analyze the differences of effects of GC16 on non-target organisms across three organisms, and an independent sample *t* test was used to compare differences of pesticides treatments (GC16 vs. Control) for the same organism. Statistical significance was set at *p* < 0.05.

## 3. Results

### 3.1. Bioassays

#### 3.1.1. Bioassays of GC16 against Different Stages of *T. pueraricola*

To evaluate the effects of GC16 against *T. pueraricola*, the LC_50_ values were determined by spraying method (Table 1). The LC_50_ values of GC16 against eggs, larvae, nymphs, and adults of *T. pueraricola* were in the range of 1.266~2.239 g/L, and the LC_90_ values ranged from 5.951 to 26.888 g/L. Results indicated that GC16 had clear insecticidal effects on all instars and stages of *T. pueraricola*, and their mortality increased with increasing concentrations of GC16; however, the same concentration of GC16 had different lethal effects on mites in different stages. At a concentration of 6.67 g/L, the mortality of female adults reached over 80% (Table S1).

#### 3.1.2. Bioassays of GC16 by Different Bioassay Method

**Figure 1:**
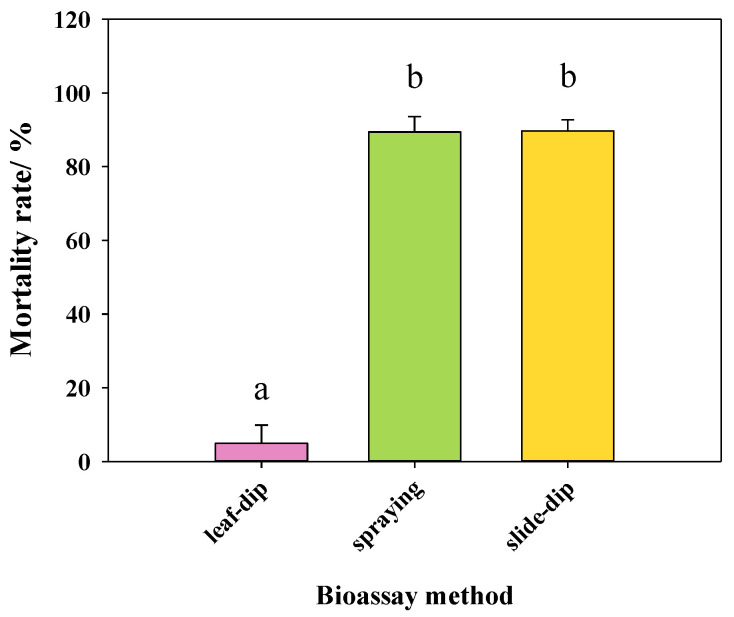
The mortality rates after 24 h of *Tetranychus pueraricola* under 6.67 g/L GC16 with different bioassay methods. Data are expressed as mean ± SE. Different lowercase letters represent significant differences at *p* < 0.05 based on a one-way ANOVA with Tukey’s HSD test.

The spray method and slide-dip method are commonly used techniques to determine the contact toxicity of pesticides, while the leaf-dip method can better test the stomach toxicity. To determine the mode of action of GC16, we compared its toxicity by different bioassay methods. The results showed that the mortality rate of *T. pueraricola* treated with 6.67 g/L GC16 by the leaf-dip method was 4.94%, while the mortality rates of the spraying method and slide-dip method were both greater than 80%, which were significantly higher than that of the leaf-dipping method (Figure 1). There was no significant difference between the spray method and the slide-dip method. The results of these experiments indicated that GC16 mainly acted on mites through contact.

#### 3.1.3. Bioassays for the Different Components of GC16

**Figure 2:**
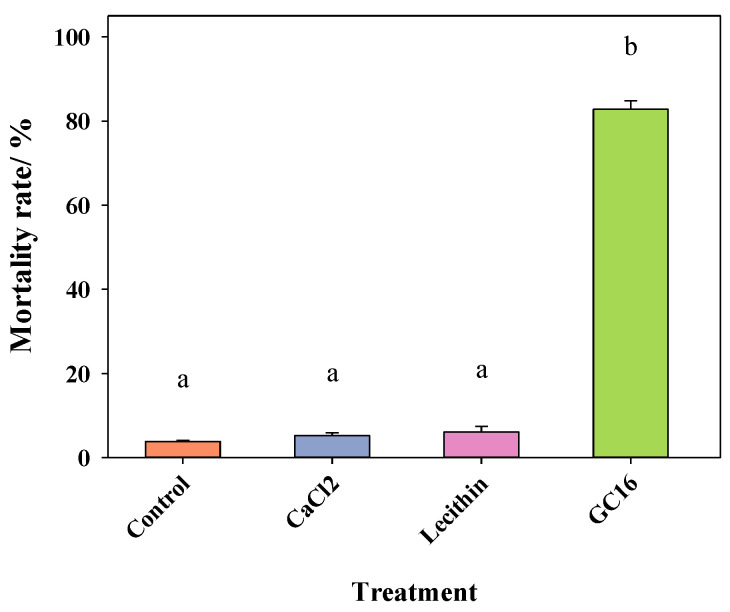
The mortality rates for 24 h of *T. pueraricola* under GC16 and its components. Component 1 is Calcium chloride (CaCl_2_), and Component 2 is Lecithin. Data are expressed as mean ± SE. Different lowercase letters represent significant differences among different treatments at *p* < 0.05 based on one-way ANOVA in Tukey’s HSD test.

GC16 is composed of a mixture of CaCl_2_ and lecithin. To determine which component is the main active component and/or how the two components synergize, the lethal efficacy of each component was tested and the results were compared. The mortality rates of mites were less than 10% when CaCl_2_ or lecithin acted alone, and there were no significant differences between control and the CaCl_2_/lecithin alone groups. However, the mortality of mites treated for 24 h with GC16 reached 80%, and the coapplication of lecithin + CaCl_2_ significantly increased mortality compared with mites treated with lecithin or CaCl_2_ alone (Figure 2). These data indicated that CaCl_2_ or lecithin alone were not lethal to mites, but the combination of lecithin + CaCl_2_ (GC16) produced very different results.

### 3.2. Observation of Poisoning Symptoms

To investigate the mechanism by which GC16 influenced *T. pueraricola,* the poisoning and death symptoms of *T. pueraricola* were observed. After GC16 treatment, the mites first entered the quiescent stage (Table 2). Then, they died and later shriveled at high concentrations. At a low concentration, some mites gradually resumed movement, while the others moved slightly and then became sluggish until death.

### 3.3. Effects of GC16 on the Morphology of Female Adult T. pueraricola

**Figure 3:**
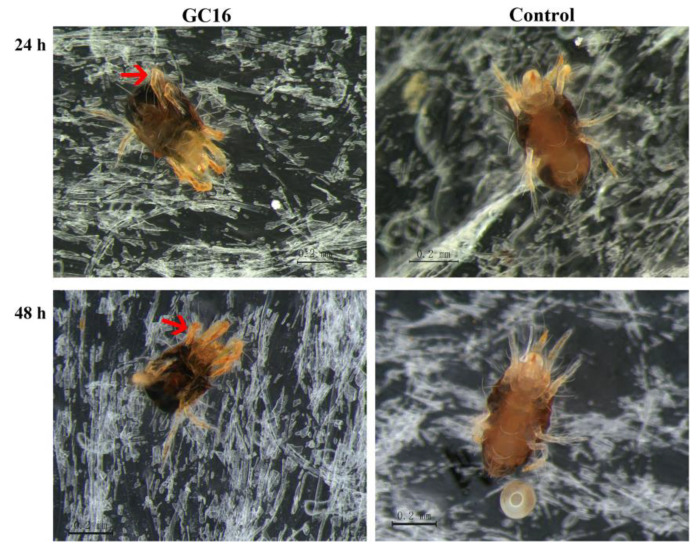
The morphology of female adults for *T. pueraricola* after GC16 treatment by slide-dip method for 24 h and 48 h. The red arrow indicates that the legs of mite were bent and curled up after treatment with GC16.

After GC16 treatment, most of the tested mites died 24 h later, but the control mites treated with water were still alive and active enough to walk, forage, and oviposit. Under a microscope, it was clearly seen that the mite bodies became small, crumpled, and shriveled and the legs became bent and curled up after treatment with GC16 (Figure 3). Compared with the water control, the body lengths of the mites treated with GC16 were significantly shortened at 24 h and 48 h, and the relative shrinkage rates were 14% and 25%, respectively (Table 3). In addition, the body widths of the mites treated with GC16 for 48 h were significantly smaller than those of the control, and the relative shrinkage rate reached 14%.

### 3.4. Effects of GC16 on the Hatching Rate and Developmental Duration of T. pueraricola Egg

**Figure 4:**
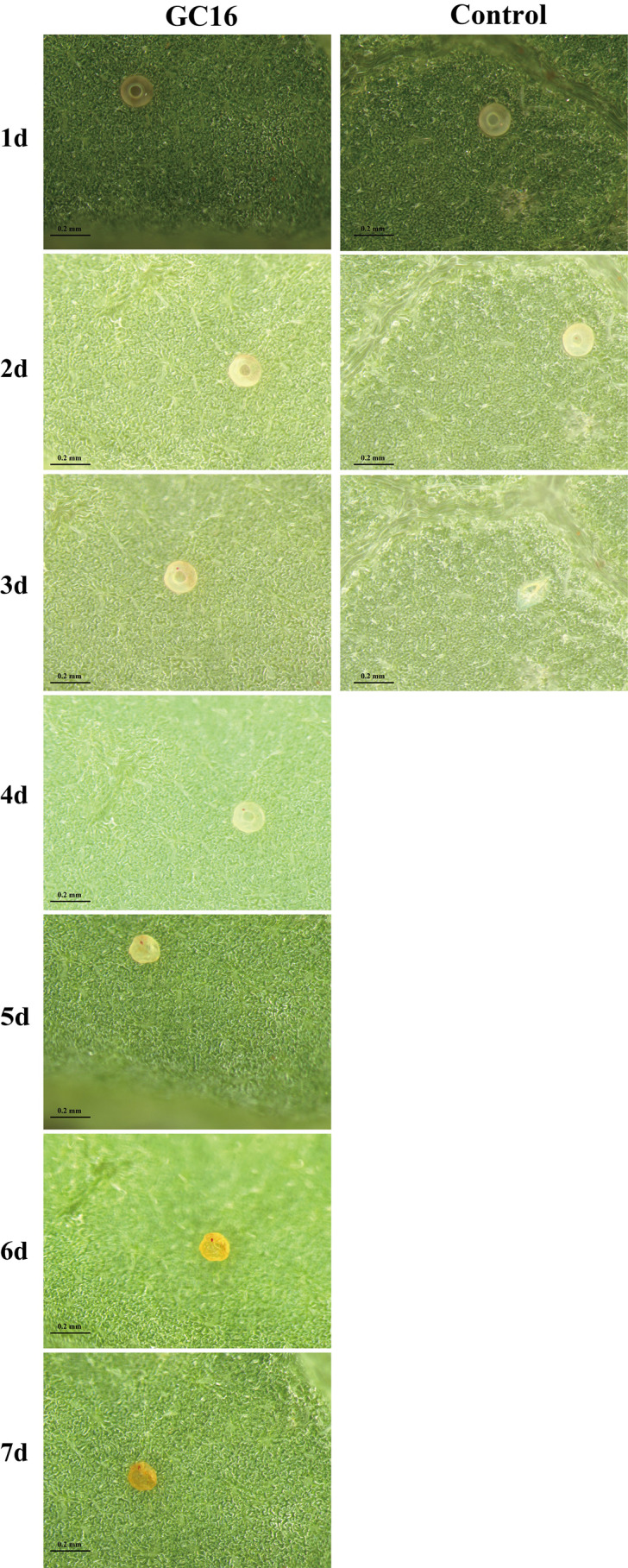
The egg morphology of *T. pueraricola* after GC16 treatment (10 g/L) vs. Control (water).

After treatment with GC16, it was found that the mite eggs gradually shriveled, became withered and deformed, and could not hatch successfully. However, under the same conditions, the eggs treated with water could molt and hatch normally (Figure 4). The hatching rates of eggs treated with GC16 and control were 14.30% and 94.38%, respectively, and there was a significant difference between them (*t* = 4.576, *df* = 4, *p* < 0.001 Table S2). Moreover, the development duration of the control eggs in water was 4.07 days, while their development duration after GC16 treatment was 5.10 days, a significant difference of 1.03 days longer than that in water (*t* = 1.480, *df* = 4, *p* = 0.005), indicating that GC16 could significantly reduce the hatching rate of eggs and prolong the egg development duration.

### 3.5. Cryo-SEM Analysis

**Figure 5:**
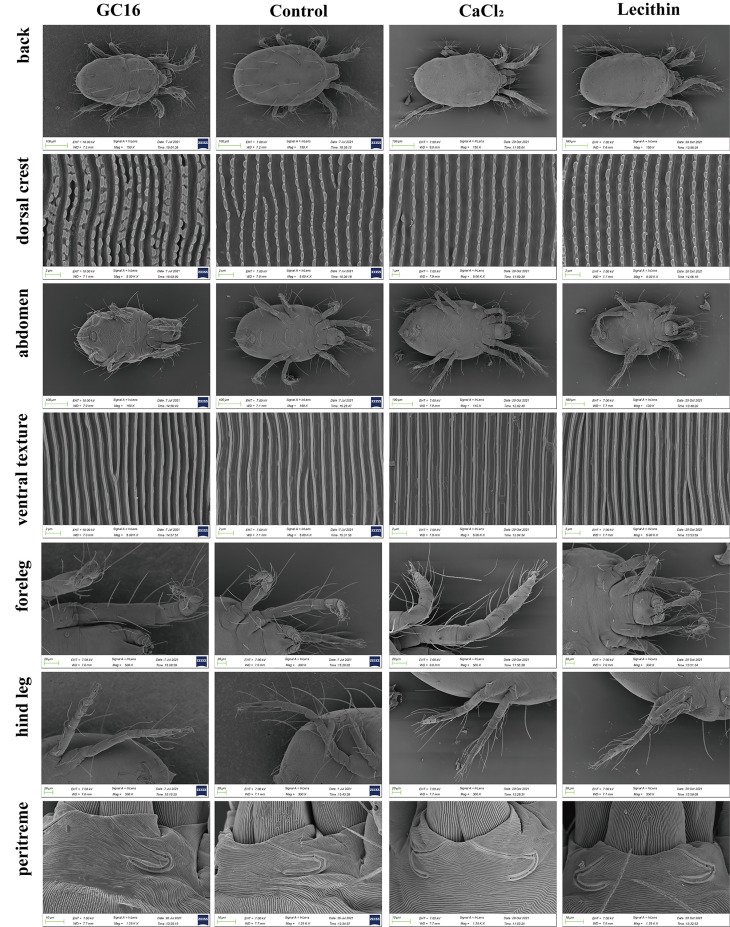
Scanning electron microscopy (SEM) observation of *T. pueraricola* after treated for 24 h. The treatments include GC16, Control (water), CaCl_2_, and lecithin. The parts that were photographed included the back, dorsal crest, abdomen, ventral texture, foreleg, hind leg, and peritreme.

According to the above results, the application of GC16 caused the insect bodies to dehydrate and atrophy; however, is this related to cuticle damage? Considering that cuticle penetration plays an important role as an insecticide mechanism, to determine the cuticle integrity of mites treated with GC16, cryo-SEM was performed. After GC16 treatment, the crest lines on the dorsal surfaces of *T. pueraricola* showed disordered and irregular arrangements, and the cuticle ridges snuggled close each other (Figure 5). In contrast, the dorsal dermatoglyphs of control (water-treated) *T. pueraricola* were arranged in an orderly and regular manner, and the cuticle ridge was evenly and regularly distributed. Additionally, there were no obvious abnormalities in the dorsal crest with CaCl_2_ or lecithin treatment alone. Furthermore, no obvious differences were seen in the forelegs, hind legs, abdomens, or peritremes between the mites receiving different treatments.

### 3.6. TEM Analysis

**Figure 6:**
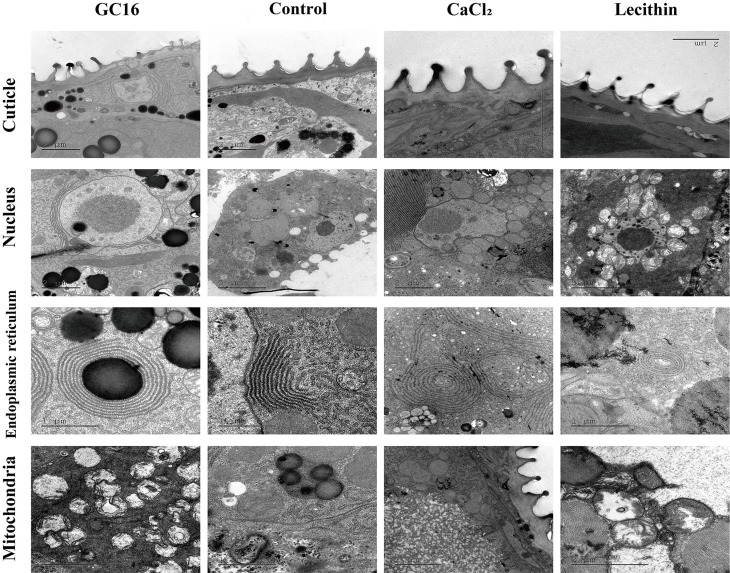
Transmission electron microscopy (TEM) observation of *T. pueraricola* after treated with GC16, Control (water), CaCl_2_, and lecithin for 24 h. The cuticle, nucleus, endoplasmic reticulum, and mitochondria of mites were photographed.

From previous poisoning symptom observations, mites displayed inactive and motionless states after GC16 treatment, and it is unclear whether this state is related to energy metabolism. To determine the cause behind these symptoms, the ultramicrostructure of the inner tissue was observed. TEM observations showed that the endoplasmic reticulum, mitochondria, and nuclear membrane system were not damaged, and the cuticle was compact and had regular protuberances in the control (water group) (Figure 6). The submicroscopic structure of the GC16 group was also clearly visible, with no abnormalities in the nucleus or endoplasmic reticulum but obvious abnormalities in the mitochondria were found. Specifically, the mitochondria were swollen and malformed, the intercristae matrix was vacuolated, and a flocculent amorphous substance appeared in the mitochondrial lumen. Compared with the water control, the cuticles of the mites treated with GC16 were dissolved, protrusion was seriously damaged, and the arrangement of the crest was irregular. Notably, there was no clear cuticle damage or organelle damage in the CaCl_2_ treatment group. In the lecithin treatment group, except for mitochondrial abnormalities (the mitochondria swelled irregularly and their cristae became fractured and fuzzy), additional changes were not noted.

### 3.7. The Effects of GC16 on the Non-Target Organisms

Safety assessment for non-target organisms is an important part of pesticide environmental toxicology, which guides agricultural production. Here, the mortality rate for mites treated with GC16 was more than 80%, while the mortality rates for non-target organisms *P. lewisi* and *H. axyridis* under GC16 treatment were less than 10% and there were no significant differences on mortality rates for them between GC16 treatment and the blank control, respectively (Table 4). This indicates that GC16 has a good control efficacy on the spider mite *T. pueraricola*, while it has no lethal effects on non-target organisms, and GC16 may have the potential to be developed as an eco-friendly acaricide.

## 4. Discussion

Exploring novel pesticides has been an important part of integrated pest management (IPM) in the current situation of agricultural development. In this study, to evaluate the performance of a new agent, GC16, on *T. pueraricola*, we carried out bioassays with mites at different developmental stages with different treatment methods; we also examined the bioassays for different components of GC16 in addition to their combination. The results showed that GC16 had effects on the eggs, larvae, nymphs, and adults of *T. pueraricola* by contact with the synergistic reaction mechanism of lecithin and CaCl_2_. Subsequently, ultrastructures of the mites were observed. The combined results demonstrated that GC16 killed mites by damaging their cuticles to first dehydrate and then destroying the mitochondria to disrupt metabolism, making the mites inactive.

In general, the median lethal concentration (LC_50_) is the elemental parameter to analyze the acaricidal activity of acaricides. The LC_50_ value is affected by the pest species, type of pesticide, bioassay method, bioassay time, and treatment environment [51,52,53]. Previous studies have reported that the LC_50_ values of avermectin, bifenazate, etoxazole, and spirodiclofen against *T. cinnabarinus* over 24 h by the slide-dip method were 3.2 × 10^−^^6^, 14.932 × 10^−3^, 4.4 × 10^−^^4^, and 0.356 g/L, respectively [52]. Additionally, the LC_50_ values of the botanical pesticide scoparone against *T. cinnabarinus* and *T. urticae* by the slide-dip method were found to be 0.279 and 0.906 g/L, respectively [53]. The LC_50_ value at 24 h of osthole to *T. urticae* was 0.332 g/L by the spraying method [2]. The LC_50_ of the crude acetone extract from *Aloe vera* L. against female adult *T. cinnabarinus* was 6.165 g/L by the slide-dip method after 24 h [51]. Herein, we found that the LC_50_ value of GC16 against female adult *T. pueraricola* was 2.00 g/L. In contrast to commercial pesticides, the LC_50_ value of GC16 was greater but at an intermediate level when compared with the plant-derived extract. Combined with the above studies, different pesticides have different acaricidal activities against different spider mite species, and GC16 has the potential to become a new acaricide compared with the plant-derived extracts.

Poison symptom investigation is the first step in understanding the mechanism of action of pesticides. To investigate the mechanism of GC16, we observed poisoning and death symptoms of *T. pueraricola*. The findings revealed that mites became stationary after GC16 treatment and then died with curly legs and shrunken and wizened bodies, which is somewhat similar to the paralyzing effects of nerve agents but without the excitement [54]. In addition, after treatment with abamectin, pyridazin, curcumin, and scopolamine, *T. cinnabarinus* showed symptoms of excitement, coma, stasis, and death [43]. There have also been other previous studies on insect poisoning symptoms. Essential oils and monoterpenes had knockdown effects on *Musca domestica* [40]. Distinct poisoning symptoms, such as extended proboscis, expanded wings, unhooked wings, extended legs, and twisted bodies, were also observed in *Apis mellifera mellifera* [42].

Naturally, insecticidal agents control insects through a variety of mechanisms, including contact, stomach, repellent, fumigant, and systemic methods or through food intake prevention or oviposition inhibition, etc. [40,49,51]. Zhang et al. (2013) found that the *A. vera* L. leaf acetone extract had contact acaricidal, repellent, fumigant, and oviposition inhibitory activities against *T. cinnabarinus* [51]. Ma et al. (2021a) reported that 1,3,4-oxadiazoles possessed excellent contact activity and weak systemic activity against *E. lanigerum* [49]. Here, a study of the mode of action demonstrated that GC16 had contact activity against *T. pueraricola*, similar to the mite contact activity of botanical extracts from *A. vera* and *Artemisia annua* [51,55]. The egg hatching inhibition and ovicidal activity of GC16 against *T. pueraricola* is consistent with azadirachtins against the maize stem borer *Chilo partellus* [56]. Moreover, egg hatching inhibition and the delay in egg hatching of *C. partellus* were supposedly due to the overall detrimental effects of azadirachtins on the reproductive systems of *C. partellus* [56]. Furthermore, scoparone was found to bind to the Vg protein and lower Vg gene expression to inhibit egg development in *T. cinnabarinus* [57]. Whether the decrease in egg hatching rate and prolongation of the developmental duration observed in this work are related to the reproductive system needs further exploration.

Undoubtedly, investigating the action mechanisms of pesticides against pests is an important strategy to develop new prospective pesticides. To explore the action mechanism, SEM, TEM, cuticle permeability, enzyme activity, gene expression profile, and RNAi are generally analyzed [49,52,53,58,59].

Cryo-SEM is an important method to study the surfaces of biological samples rich in water. Compared with traditional SEM, there is no sample pretreatment processes required for cryo-SEM, which would inevitably be related to sample distortion, shrinkage, or a loss of the inner cellular soluble components; therefore, we can obtain the most realistic images of sample shape and structure [46,60]. In this work, through cryo-SEM, we found that the cuticle layer of *T. pueraricola* was destroyed and its arrangement was disordered by GC16, similar to another finding, i.e., graphene oxide can absorb and impair the structure of the cuticle layer of mites [52]. The insect cuticle is its primary protective barrier against the penetration of pesticides. Previous studies have shown that pesticides more easily penetrate weaker and damaged cuticles, and cuticle damage is positively correlated with insecticide permeability and insect mortality [61,62]. Moreover, cuticle permeability is regarded to be associated with insecticide sensitivity, and damaged cuticles are often accompanied by dehydration and shriveling [52,58]. Therefore, the reason for death of *T. pueraricola* in this study might be that the impaired cuticle layer lost its protective function against the penetration of GC16.

In terms of TEM, ultrastructural changes were detected in *T. pueraricola*, indicating that GC16 might exert its acaricidal activity by destroying the mitochondria and perturbing the cuticle layer array. It has been previously reported that the steroid PSNW targets the midgut cells of *Mythimnazus separata* Walker by destroying the cell membrane and mitochondria [63]. In addition, the target site of the steroid 1,3,4-oxadiazole was demonstrated to be the mitochondria and nucleus in the midgut tissues of *Eriosoma lanigerum* [49]. Mitochondria are the powerhouses of the cell and mitochondrial dysfunction is related to oxidative homeostasis and lipid and energy metabolism [50,64]. For example, zebrafish exposed to triazoles had impaired mitochondrial oxidative phosphorylation and oxidative stress as well as dysregulation of lipid metabolism, which resulted in developmental disorders and movement disorders [64]. In this study, we found mitochondrial dysfunction in mites treated with GC16 accompanied by motionless poisoning symptoms, which were inferred to be related to lipid or energy dysmetabolism. In addition, the mitochondrial dysfunction phenomenon of mites after GC16 treatment was similar to treatment with cyflumetofen, which was demonstrated to be an inhibitor of complex II in the mitochondrial electron transport chain [65]. Mitochondrial abnormalities were also seen in mites in the lecithin group; however, the corresponding mortality rate of the mites after this treatment was low, which might be because lecithin acted alone with a sublethal effect rather than a lethal effect. It was also previously reported that lecithin could induce mitochondrial membrane alterations in mammals but lecithin effectively protected certain sperm quality characteristics against freezing-induced damage [66]. Lecithin is structurally similar to the cell membrane (both contain phospholipids). Moreover, the epicuticle is the outermost layer of the insect integument and mainly composed of lipids and proteins. According to the principle of “like dissolves like”, lecithin can dissolve cell membranes and the cuticles of insects in theory. In addition, calcium chloride has the property of water absorption and may influence Ca^2+^ balance of the mite. Therefore, we speculate that it is the symmetrical structure of GC16 [inorganic ions + organic substance (dissolve membrane)] that caused the cuticle of mite to be adsorbed, dissolved, and lose water and to lead to ionic imbalance.

Overviewing the action mechanism of pesticides, the Ca^2+^ homeostasis disruption and cuticle permeability increase hypotheses were mentioned. For example, Zhou et al. (2021a) reported that curcumin might activate and overexpress the CaM gene and disrupt Ca^2+^ homeostasis in *T. cinnabarinus* to achieve the control effect [67]. Scopoletin acts by regulating the calcium signaling pathway and disrupting intracellular Ca^2+^ homeostasis [68]. Further results showed that the acaricidal mechanism of scopoletin on *T. cinnabarinus* may be related to the calcium channel gene TcT-VDCC [69]. In addition, the mechanism of action of scoparone against *T. cinnabarinus* is by targeting the interface between CaM1 and L-VGCC to activate the CaM binding site located in the IQ motif at the L-VGCC C-terminus [53]. Moreover, scopoletin could act on mites by inhibiting chitinase (CHIT) gene expression [70], and graphene oxide could inhibit the expression of the cuticle protein (CPR) gene to disturb the construction of the cuticle layer and increase cuticle permeability and acaricide sensibility [52]. Additionally, in *Blattella germanica*, low expression of CYP4G19 disordered the array of the lipid layer, enhanced cuticle permeability, and compromised insecticide tolerance [58]. Because CaCl_2_ is an important component of GC16, whether the application of GC16 affects the calcium homeostasis in mites needs further exploration. However, the destruction of the cuticles of the mites in this study supports the cuticle penetration hypothesis.

Putting the above together, GC16 exhibited a stronger lethal effect on *T. pueraricola* than lecithin or CaCl_2_ alone. In addition, GC16 destroyed and disordered the cuticles of the mites, while those treated with lecithin or CaCl_2_ alone had intact and regular cuticles. In contrast to the normal mitochondria of the control and CaCl_2_ group, there were mitochondrial abnormalities, such as inner ridge fracture and degradation, in the GC16 group and lecithin group. The combined results suggest that GC16 broke the cuticles of the mites by the coapplication of lecithin + CaCl_2_ and lecithin was the main source of *T. pueraricola* mitochondria damage, indicating that cuticle damage was more important than mitochondrial dysfunction for the lethal effects of GC16 against mites.

## 5. Conclusions

In conclusion, we found that GC16 caused insecticidal efficacy against *T. pueraricola* through contact by disordering the arrangement of the crest in the cuticular layer and destroying the mitochondria. Considering that 6.67 g/L GC16 (recommended concentration for the control of *T. pueraricola*) has no lethal effects on natural enemy insects *P. lewisi* and *H. axyridis*, it may possess the potential to be developed as an ecological agent. However, the effects of GC16 on the overall ecosystem need to be further evaluated in field applications. Our findings may accelerate the development of novel ecological pesticides to control destructive spider mites worldwide. Furthermore, this work will provide alternative pesticide support for pest control for sustainable agriculture; for example, planting of Chinese medicinal herbs. Moreover, how GC16 acts on the cuticle and mitochondria and which genes are involved in this process remain to be studied.

## Figures and Tables

**Table 1:** The overall lethal effect of GC16 on *Tetranychus pueraricola* in the different developmental stages.

Stage	N ^a^	Slope ± SE	LC_50_ (95% FL ^b^(g/L))	LC_90_ (95% FL ^b^(g/L))	χ2(df) ^c^
egg	425	1.187 ± 0.182	2.239 (1.349 ~ 3.131)	26.888 (16.980 ~ 58.918)	2.615 (10)
larva	885	2.652 ± 0.202	1.963 (1.655 ~ 2.300)	5.973 (4.759 ~ 8.245)	21.988 (13)
nymph	686	1.907 ± 0.199	1.266 (0.782 ~ 1.705)	5.951 (4.044 ~ 12.833)	39.524 (13)
adult	625	2.107 ± 0.193	1.996 (1.624 ~ 2.396)	8.099 (6.143 ~ 12.155)	17.138 (13)

^a^ Number of adult mites used in the bioassay, including controls. ^b^ FL = fiducial limit. ^c^ Chi-square value and degrees of freedom.

**Table 2:** Poisoning symptoms after the GC16 treatment.

Treatment (g/L)	20 min	40 min	2 h	4 h	6 h	8–18 h	18–24 h
Control (0)	feed and oviposit actively	feed and oviposit actively	feed and oviposit actively	feed and oviposit actively	feed and oviposit actively	feed and oviposit actively	feed and oviposit actively
GC16 (2.00)	stationary	stationary	moved slightly	moved slightly	1. crawled 2. moved slightly 3. stationary/died	1. crawled 2. moved slightly 3. died	1. crawled 2. wiggled when tapped 3. died
GC16 (6.67)	stationary	stationary	stationary	stationary/died	stationary/died	died, started shrinking	shriveled

Note: 2.00 g/L (LC_50_) is the concentration of GC16 lethal to 50% of adult mites, while 6.67 g/L, the recommended concentration for field use, is the concentration of GC16 lethal to approximately 85% of the adult mites.

**Table 3:** Mean (± SE) body length and width of female adult *T. pueraricola* under GC16 treatment.

Treatment	Body Length (mm)	Rst for Body Length	Body Width (mm)	Rst for Body Width
Control-24 h	0.57 ± 0.01 ^a^	24 h Rst = 14.36%	0.28 ± 0.01 ^a,b^	24 h Rst = −0.16%
GC16-24 h	0.49 ± 0.01 ^b^		0.28 ± 0.01 ^a,b^	
Control-48 h	0.56 ± 0.01 ^a^	48 h Rst = 24.69%	0.30 ± 0.01 ^a^	48 h Rst = 14.53%
GC16-48 h	0.42 ± 0.01 ^c^		0.26 ± 0.01 ^b^	

Note: Rst indicates the relative shrinkage rate. Control/GC16-24 h/48 h indicates the mites were treated with water/GC16 for 24 h/48 h, respectively. Data are expressed as mean ± SE. Different lowercase letters in the same column indicate significant differences between treatments (*p* < 0.05). One-way ANOVA and Tukey’s honestly significant difference (HSD) test were adopted. The *F* and *p* values for HSD test of body length and body width are *F*(3,20) = 18.431, *p* < 0.001 and *F*(3,20) = 2.972, *p* = 0.056, respectively.

**Table 4:** The mortality rates (Mean ± SE) of target organism (*Tetranychus pueraricola*) and non-target organisms (*Picromerus lewisi, Harmonia axyridis*) after treating with GC16 vs. Control (water).

Organism	Mortality Rate (%)		*t*, *p*
	GC16	Control	
*Tetranychus pueraricola*	86.21 ± 2.02 ^A,a^	2.38 ± 1.19 ^A,b^	*t* = −35.693, *df* = 4, *p* < 0.001
*Picromerus lewisi*	4.15 ± 0.23 ^B,a^	4.35 ± 0.37 ^A,a^	*t* = 0.447, *df* = 4, *p* = 0.678
*Harmonia axyridis*	8.33 ± 1.67 ^B,a^	5.83 ± 0.83 ^A,a^	*t* = −1.342, *df* = 4, *p* = 0.251

Note: Values are mean ± SE. The same uppercase letter in a column expresses no significant difference among three organisms according to the one-way ANOVA and Tukey’s honestly significant difference (HSD) test, and the same lowercase letter in a row indicates no significant difference between GC16 treatment (6.67 g/L) and control (water) based on independent sample *t* test at *p* < 0.05. The *F* and *p* values for HSD test of GC16 and Control across the three organisms are *F*(2,6) = 925.992, *p* < 0.001 and *F*(2,6) = 3.973, *p* = 0.080, respectively.

## Data Availability

The data presented in this study are available in article or Supplementary Materials.

## Supplementary Materials

The following supporting information can be downloaded at: https://www.mdpi.com/article/10.3390/insects13050433/s1, Table S1: The raw data of bioassays of GC16 on mites, Table S2: The egg hatchability and developmental duration of *T. pueraricola* under GC16 treatment.
