# Supplementary material for: The Insecticidal Efficacy and Physiological Action Mechanism of a Novel Agent GC16 against Tetranychus pueraricola (Acari: Tetranychidae)
Source: Insects. 2022 May 5;13(5):433. doi: 10.3390/insects13050433 (PMC9146473; doi:10.3390/insects13050433)
Supplement: Supplementary file 1 [file insects-13-00433-s001.zip › insects-1691313-supplementary.pdf]

Table S1. The raw data of bioassays of GC16 on mites

Stage	Concentration (g/L)	All[a]	24 h-D[b]	24 h-L[c]
egg	0	92	4	88
	1.25	80	32	48
	5	84	56	28
	10	99	82	17
	20	70	59	11
larva	0	165	14	151
	0.83	143	31	112
	1.67	213	113	100
	3.33	91	56	35
	6.67	97	90	7
	13.33	176	176	0
nymph	0	93	6	87
	0.83	108	53	55
	1.67	189	109	80
	3.33	110	81	29
	6.67	98	93	5
	13.33	88	88	0
adult	0	109	6	103
	0.83	118	34	84
	1.67	118	50	68
	3.33	109	79	30
	6.67	92	75	17
	13.33	79	79	0

Note: a, Number of all tested mites. b, Number of all died mites in 24 h after GC16 treatment. c, Number of all live mites in 24 h after GC16 treatment.

Table S2. The egg hatchability and developmental duration of *T. pueraricola* under GC16 treatment

Treatment	Hatching rate (%)	Mean ± SE (%)	Duration (day)	Mean ± SE (day)
Control	87.50	94.38 ± 3.66 a	4.380	4.070 ± 0.160 b
Control	100.00		3.875	
Control	95.65		3.950	
GC16	15.56	14.30 ± 0.73 b	5.000	5.100 ± 0.097 a
GC16	14.29		5.000	
GC16	13.04		5.290	
t, df, P	$t = 4.576$, $df = 4$, $p < 0.001$		$t = 1.480$, $df = 4$, $p = 0.005$	

Note: The concentration of GC16 was 10 g/L. Independent sample *t* test was used for one way ANOVA, and different lowercase letters in the same column indicated significant difference between treatments ($p < 0.05$).
